# Localities of Life

**Published:** 1874-11

**Authors:** 


### LOCALITIES OF LIFE.

The mountain top is healthier than the valley because the air is purer, made so by the constant renewal of the winds, and by its exemption from impregnation and saturation with the effluvia arising from decaying refuse of vegetable and animal matters.

Elevated situations are generally exempt from the ravages of consumptive disease. One in a hundred of all the deaths in the city of Mexico, which is ten thousand feet above the level of the sea, is the result of common consumption of the lungs, whereas in our seaboard cities, not fifty feet above the surface of the water, the proportion is twenty in a hundred. The prominent physiological reason for this is that the air is lighter, contains less oxygen; but as the lungs live on oxygen, as it is the oxygen which they bring in contact with the blood at every breath, it is that which purifies it and gives to it its life-giving power. One result is inevitable; each breath of air not giving a sufficient amount of oxygen, instinct prompts a deeper breath, a fuller breath; the lungs are compelled to make greater efforts to take in a greater breath of air, this distends them more fully, the blood vessels on the sides of the air cells are straightened out, hence a want of a free circulation of the contained fluid, and a free circulation of the blood is an essential element of health.

Many physicians have advised systematic and forcible and deep inspirations as a means of filling out and developing the lungs, doubtless to great advantage.

The lungs always begin to fail at the top, just under the collar bone. In examining persons after death from consumption, the lungs will be found decayed at the top, three times out of four. In process of taking air into the lungs, the lower portion is first distended, as in filling a bladder, and the upper part last. In the ordinary breathing of those who are most of the time in the house, only the lower portion of the lungs is filled with air, as the breath is not drawn in with sufficient force to fill the upper part, hence this upper part is soon attacked with consumption. Therefore, whatever course of life is pursued which promotes full and deep inspirations, most promotes the arrest and cure of consumptive disease. And, as in the event of disease, the lungs do not take in as much air as is needed for the wants of the system, what is breathed should be the purest possible, and that is out-door air ; therefore those who are out of doors most, whether day or night, hot or cold, stand the best chance of getting well ; in fact, there is no hope of warding off consumption that is threatened, much less of curing it, without a free employment of out-door activities for a great part of every day. A consumptive who "camps out," even in winter, is far more likely to get well than one who remains indoors, keeping his room at the same degree of temperature all the time.

It may be of interest to know the elevation and population in round numbers of some of the principal cities of the Union :

| Name. | Elevation. | Population. |
|---|---|---|
| New Orleans, - | 10 | 200,000 |
| New York, - | 35 | 1,000,000 |
| Philadelphia, - | 35 | 750,000 |
| Brooklyn, - | 40 | 425,000 |
| Boston, - | 40 | 275,000 |
| Washington, - | 45 | 150,000 |
| San Francisco, - | 50 | 200,000 |
| Baltimore, - | 60 | 300,000 |
| St. Louis, - | 475 | 350,000 |
| Cincinnati, - | 575 | 275,000 |
| Chicago, - | 585 | 400,000 |
| Delaware, O., - | 1,000 | 6,000 |
| Canton, O., - | 1,000 | 10,000 |
| Atchison, Kansas, | 1,000 | 8,000 |
| Meadville, Pa., - | 1,000 | 7,000 |
| Knoxville, Tenn., | 1,000 | 10,000 |
| Nebraska,City,Neb., | 1,000 | 7,000 |
| Madison, Wis., - | 1,050 | 10,000 |
| Titusville, Pa,, - | 1,050 | 10,000 |
| Atlanta, Ga., - | 1,050 | 30,000 |
| Corry, Pa., - | 1,100 | 7,000 |
| Hudson, N. Y., - | 1,100 | 9,000 |
| Mansfield, O., - | 1,140 | 9,000 |
| Mahoning, Pa., - | 1,200 | 6,000 |
| Johnstown, Pa., - | 1,200 | 5,000 |
| Addison, Mich., - | 1,200 | 9,000 |
| Council Bluffs, Iowa, | 1,200 | 1,1000 |
| Altoona, Pa., - | 1,220 | 11,000 |
| Staunton, Va., - | 1,350 | 6,000 |
| Jamestown, N. Y., | 1,350 | 6,000 |
| Springfield, Mass., | 1,360 | 7,000 |
| Winona, Miss., - | 1,500 | 10,000 |

